# The Monte Carlo Simulation of Three Antimicrobials for Empiric Treatment of Adult Bloodstream Infections With Carbapenem-Resistant Enterobacterales in China

**DOI:** 10.3389/fmicb.2021.738812

**Published:** 2021-11-25

**Authors:** Dongna Zou, Guangyue Yao, Chengwu Shen, Jinru Ji, Chaoqun Ying, Peipei Wang, Zhiying Liu, Jun Wang, Yan Jin, Yonghong Xiao

**Affiliations:** ^1^Department of Pharmacy, Shandong Provincial Hospital Affiliated to Shandong First Medical University, Jinan, China; ^2^Cancer Therapy and Research Center, Shandong Provincial Hospital Affiliated to Shandong University, Jinan, China; ^3^State Key Laboratory for Diagnosis and Treatment of Infectious Diseases, College of Medicine, The First Affiliated Hospital, Zhejiang University, Hangzhou, China; ^4^National Clinical Research Center for Infectious Diseases, College of Medicine, The First Affiliated Hospital, Zhejiang University, Hangzhou, China; ^5^Collaborative Innovation Center for Diagnosis and Treatment of Infectious Diseases, The First Affiliated Hospital, College of Medicine, Zhejiang University, Hangzhou, China; ^6^Department of Clinical Laboratory, Shandong Provincial Hospital Affiliated to Shandong First Medical University, Jinan, China

**Keywords:** bloodstream infections, carbapenem-resistant Enterobacteriaceae, polymyxin B, ceftazidime/avibactam, tigecycline, Monte Carlo simulation

## Abstract

**Introduction:** The aim of this study was to predict and evaluate three antimicrobials for treatment of adult bloodstream infections (BSI) with carbapenem-resistant Enterobacterales (CRE) in China, so as to optimize the clinical dosing regimen further.

**Methods:** Antimicrobial susceptibility data of blood isolates were obtained from the Blood Bacterial Resistance Investigation Collaborative Systems in China. Monte Carlo simulation was conducted to estimate the probability target attainment (PTA) and cumulative fraction of response (CFR) of tigecycline, polymyxin B, and ceftazidime/avibactam against CRE.

**Results:** For the results of PTAs, tigecycline following administration of 50 mg every 12 h, 75 mg every 12 h, and 100 mg every 12 h achieved > 90% PTAs when minimum inhibitory concentration (MIC) was 0.25, 0.5, and 0.5 μg/mL, respectively; polymyxin B following administration of all tested regimens achieved > 90% PTAs when MIC was 1 μg/mL with CRE; ceftazidime/avibactam following administration of 1.25 g every 8 h, 2.5 g every 8 h achieved > 90% PTAs when MIC was 4 μg/mL, 8 μg/mL with CRE, respectively. As for CFR values of three antimicrobials, ceftazidime/avibactam achieved the lowest CFR values. The highest CFR value of ceftazidime/avibactam was 77.42%. For tigecycline and ceftazidime/avibactam, with simulated regimens daily dosing increase, the CFR values were both increased; the highest CFR of tigecycline values was 91.88%. For polymyxin B, the most aggressive dosage of 1.5 mg/kg every 12 h could provide the highest CFR values (82.69%) against CRE.

**Conclusion:** This study suggested that measurement of MICs and individualized therapy should be considered together to achieve the optimal drug exposure. In particular, pharmacokinetic and pharmacodynamic modeling based on local antimicrobial resistance data can provide valuable guidance for clinicians for the administration of empirical antibiotic treatments for BSIs.

## Introduction

Bacterial drug resistance is becoming more and more serious. The monitoring of drug-resistant bacteria and the management of antimicrobials have valued more attention from all over the world. Carbapenems are the most potent β-lactam family of antibiotics for the treatment of bacterial infections, especially Enterobacteriaceae infections (Rahal, 2008), and are regarded as the “last resort” in the treatment of Gram-negative bacterial infections (El-Gamal et al., 2017). Once strains are resistant to carbapenem, the treatment will face great difficulties.

However, in the past few decades, the isolation of carbapenem-resistant Enterobacterales (CRE) strains has greatly increased, which bring great difficulties and challenges in clinical treatment. In many countries in the world, such as Europe, Asia, South America, and North America, outbreaks caused by CRE have been reported. CRE has become a global public health threat now (Sievert et al., 2013). The US Centers for Disease Control and Prevention (CDC) also lists CRE as a threat to public health in 2015 (Centers for Disease Control and Prevention, 2013). According to the US CDC, the incidence of CRE increased from 1.2% in 2001 to 4.2% in 2011 (Little et al., 2012). Chen et al. (2021) reported that in a population-based study in seven states in the United States, CRE incidence was up to 2.93 per 100,000 persons. The complex resistance mechanisms have also brought more troubles to treatment, especially bloodstream infections (BSIs) with CRE, which have been rapidly spreading worldwide with a high mortality and pose a challenge to therapeutic decision-making (Tumbarello et al., 2012; Laupland and Church, 2014; Wu et al., 2020). As the most serious type of infections caused by CRE, BSI usually leads to a worse prognosis, longer hospital stay, and higher mortality (Neuwirth et al., 1995; Hussein et al., 2013). The fatality rate of patients with CRE infections was significantly different in different studies; the fatality rate of BSIs is 40–50% (Patel et al., 2008). According to the reports reported in the United States, Italy, Greece, and Spain, the mortality of CRE BSIs was 40–60% (Meatherall et al., 2009), and the fatality rate of BSIs in the population of neutropenia and hematological malignancies was as high as 69% (Satlin et al., 2013). Falagas et al. (2014) reported that their pooled analysis of the nine studies (985 patients) showed that the death rate was higher among CRE-infected than carbapenem-susceptible Enterobacterales (CSE)–infected patients. CRE-infected patients had an unadjusted number of deaths twofold higher than that for CSE-infected patients (Falagas et al., 2014). Compared with CSE, effective anti-infective treatment is often delayed because of the limited treatment of infections caused by CRE (Little et al., 2012), so the mortality of patients whose infections are caused by CRE is higher (Satlin et al., 2016; Averbuch et al., 2017).

The treatment of CRE infections is difficult, and the prognosis is poor; it brings great challenges to clinical treatment and nosocomial infection control. Previous study has been demonstrated that insufficient empirical antimicrobial therapy is independently associated with higher mortality in CRE BSIs (Tumbarello et al., 2012), especially in patients with inadequate initial dosing (Zarkotou et al., 2011). Thus, early administration of appropriate empirical antimicrobial therapy for BSIs with CRE is particularly important. Inappropriate antimicrobial therapy of CRE sensitive drugs may increase the selective pressure of antibacterial and increase the waste of medical resources (Dautzenberg et al., 2015; Lee and Lee, 2016). For critically ill patients, combining local pathogenic characteristics, drug sensitivity, and pharmacokinetic (PK) and pharmacodynamic (PD) characteristics of antimicrobial can improve the success rate of treatment.

To choose an optimal antibiotic or dosing regimen, susceptibility results, PK/PD factors, infection site, and patient factors (allergies or intolerances) should be considered to make an individualized treatment (Vasoo et al., 2015; Zhu et al., 2020). The combined use of the distributions of location-specific minimum inhibitory concentrations (MICs), different antibiotic regimens, and PK parameters derived from human studies via the application of PK/PD models with Monte Carlo simulation is a useful approach for predicting treatment outcomes (Bradley et al., 2003).

We examined the MIC distributions of CRE isolated from blood cultures of adults with BSIs from the Blood Bacterial Resistance Investigation Collaborative Systems (BRICS) in China, 2018–2019, as a basis for PK/PD modeling. We predicted and evaluated three antimicrobials (tigecycline, polymyxin B, and ceftazidime/avibactam) used to treat CRE-infected BSIs so as to identify the most appropriate antibiotics and dosage regimens for the empirical treatment of CRE-infected BSIs and to optimize the clinical dosing regimen further.

## Materials and Methods

### Antimicrobials

Three antimicrobials and eight dosage regimens were selected for modeling, based on their common use for the treatment of CRE-infected BSIs in China (Table 1).

**TABLE 1:** Antibiotic regimens used in the Monte Carlo simulations.

Antibiotic	Dose
Tigecycline	50 mg every 12 h
	75 mg every 12 h
	100 mg every 12 h
Polymyxin B	1.25 mg/kg every 12 h
	1.5 mg/kg every 12 h
	2.5 mg/kg per day continuous infusion
Ceftazidime/avibactam	1.25 g every 8 h
	2.5 g every 8 h

### Bacterial Isolates

The data in the present study were from the National Bloodstream Infection BRICS platform in China (50 hospitals) for 2018 and 2019. Most of the hospitals included were the largest hospitals in each province. Six hundred fifty-three non-duplicate CRE species were isolated from blood cultures. Each laboratory of the 50 hospitals identified the species using standard biochemical methodology with an automated system (Vitec 2, bioMérieux, France; MicroScan walkAway-96, Siemens, United States; or Phoenix-100, BD, United States).

### Minimum Inhibitory Concentration Determination

The MICs of tigecycline, polymyxin B, and ceftazidime/avibactam were determined by broth microdilution method or one of the three automated systems in accordance with the Clinical Laboratory Standards Institute (CLSI, 2019) guidelines.

### PK/PD Model

All the PK data were obtained from previously published studies of infected and/or critically ill patients who had adequate renal function, shown in Table 2.

**TABLE 2:** Pharmacokinetic parameters (means ± SDs) used in the Monte Carlo simulations.

Antibiotic	Cl_T_ (L/h)	Fu (%)	Vd (L)	References
Tigecycline	19.2 ± 7.76	—	—	Rubino et al., 2010
Polymyxin B	2.5 ± 0.4	—	—	Thamlikitkul et al., 2016
Ceftazidime/avibactam	7.53 ± 1.28	90	18.8 ± 6.54	Bensman et al., 2017

*Cl_T_, total body clearance; fu, fraction unbound; SDs, standard deviations; Vd, volume of distribution.*

PD exposures were simulated as free drug (f) for ceftazidime/avibactam and as total drug for tigecycline and polymyxin B.

For the tigecycline and polymyxin B, PK exposures were measured by 24-h area under the curve (AUC_24_)/MIC > 6.96 and AUC/MIC ≥ 50, respectively, to be predictive of the clinical and microbiologic efficacy (Miglis et al., 2018; Wang et al., 2020). The steady-state AUC from 0 to 24 h (AUC_0–24 h_) was calculated according to the following equation: AUC_0–24_ = dose/Cl_T_.

For ceftazidime/avibactam, PK exposures was measured by 50% fT > MIC (Wang et al., 2020), which was calculated using the following one-compartment intravenous infusion equation (Drusano et al., 2001). fu is the fraction of unbound drug, Vd is the volume of distribution in liters at steady state, MIC is the MIC, Cl_T_ is total body clearance, and DI is dosing interval.


$$\%\text{fT}>MIC=ln\left(\frac{D\,o\,s\,e\times f\,u}{V\,d\times M\,I\,C}\right)\times \frac{V\,d}{C\,L\,t}\times \frac{100}{D\,I}$$

### Monte Carlo Simulations

A 10,000-subject Monte Carlo simulation (Oracle Crystal Ball; version 11.1.2.4.400) was conducted for each antimicrobial regimen. PK data in the “PK/PD Model” section were used to determine the percentages of PK/PD target attainment (PTA) for a range of MICs from 0.03 to 64 mg/L. The probability of PTA, which represented the likelihood that an antimicrobial regimen will meet or exceed the target at a specific MIC, was assessed for each regimen. The cumulative fraction of response (CFR), which represented the expected population PTA for a specific drug dose and a specific population of microorganisms, was calculated for MIC distributions using weighted summation and calculated as follows (Drusano et al., 2001). A regimen that achieved more than 90% CFR against a population of organisms was considered optimal (Mouton et al., 2005).


$$\text{CFR}=\sum_{i=0}^{n}P\,T\,A\,i\times F\,i$$

## Results

### The Results of Susceptibility Testing

There were 653 non-duplicate CRE species isolated from blood cultures enrolled in our study during 2018 and 2019, including carbapenem-resistant *Klebsiella pneumoniae* (CRKP) (*n* = 511), carbapenem-resistant *Escherichia coli* (CREC) (*n* = 83), and other CRE species except CRKP and CREC (*n* = 59).

We analyzed the MIC data for all CRE and established discrete MIC distributions for each population based on MIC frequencies. Tables 3, 4 show the 50% MIC (MIC_50_) and 90% MIC (MIC_90_) percentage of isolates by MIC for each antimicrobial agent.

**TABLE 3:** MIC distributions for antimicrobials against all CRE isolated from blood specimens in China during 2018–2019.

MIC (mg/L)	No.^a^	Percentages of isolates by MIC												MIC_50_	MIC_90_	MIC range
		
Antibiotic		0.03	0.06	0.125	0.25	0.5	1	2	4	8	16	32	64			
CRE (*n* = 653)																
Tigecycline	646	1.39	1.86	10.22	39.16	20.12	21.83	4.49	0.62	0.31	0	0	0	0.25	1	0.03–8
Polymyxin B	650	0	0	0	4.77	54.31	22.92	12.15	2.15	1.08	1.38	1.23	0	0.5	2	0.25–32
Ceftazidime/avibactam	445	0	0.22	0.22	0.67	2.02	4.72	9.66	26.74	30.79	1.8	22.02	1.12	8	16	0.06–32

*MIC, minimum inhibitory concentration; CRE, carbapenem-resistant Enterobacterales; MIC_50_, 50% minimum inhibitory concentration; MIC_90_, 90% minimum inhibitory concentration. ^a^No., number of isolates in which antibiotic sensitivity was tested.*

**TABLE 4:** MIC distributions for antimicrobials against all CRE isolated from blood specimens in China during 2018–2019.

MIC (mg/L)	No.^a^	Percentages of isolates by MIC												MIC_50_	MIC_90_	MIC range
		
Antibiotic		0.03	0.06	0.125	0.25	0.5	1	2	4	8	16	32	64			
CRKP (*n* = 511)																
Tigecycline	511	0.98	1.57	9.59	33.86	22.31	25.64	4.89	0.78	0.39	0	0	0	0.5	1	0.03–8
Polymyxin B	511	0	0	0	4.11	57.73	20.94	11.35	2.15	0.98	1.76	0.98	0	0.5	–	0.25–32
Ceftazidime/avibactam	325	0	0.31	0.31	0	1.85	5.23	12.62	34.15	37.23	0.92	7.38	0	4	16	0.06–32
CREC (*n* = 83)																
Tigecycline	83	4.82	4.82	15.66	61.45	4.82	6.02	2.41	0	0	0	0	0	0.25	0.5	0.03–2.41
Polymyxin B	83	0	0	0	7.23	53.01	21.69	15.66	2.41	0	0	0	0	0.5	2	0.25–4
Ceftazidime/avibactam	61	0	0	0	0	3.28	3.28	1.64	6.56	24.59	6.56	54.1	0	32	32	0.5–54.1
CRE species except CRKP and CREC (*n* = 59).																
Tigecycline	52	0	0	8.93	55.36	23.21	8.93	3.57	0	0	0	0	0	0.25	1	0.125–2
Polymyxin B	56	0	0	0	6.67	28.33	41.67	13.33	1.67	3.33	0	5	0	1	2	0.25–32
Ceftazidime/avibactam	59	0	0	0	5.08	1.69	3.39	1.69	6.78	1.69	1.69	69.49	8.47	32	32	0.25–64

*MIC, minimum inhibitory concentration; CRE, carbapenem-resistant Enterobacterales; MIC_50_, 50% minimum inhibitory concentration; MIC_90_, 90% minimum inhibitory concentration;*

*^a^No., number of isolates in which antibiotic sensitivity was tested; CRKP, carbapenem-resistant Klebsiella pneumoniae; CREC, carbapenem-resistant Escherichia coli.*

For tigecycline, the MIC_50_ and MIC_90_ against CRKP, which was the strain with the highest detection rate among all CREs, were 0.5 and 1 mg/L, whereas the value of MIC_50_ and MIC_90_ were 0.5 and 2 mg/L for polymyxin B, and 4 and 16 mg/L for ceftazidime/avibactam.

### Probability Target Attainment

**FIGURE 1:**
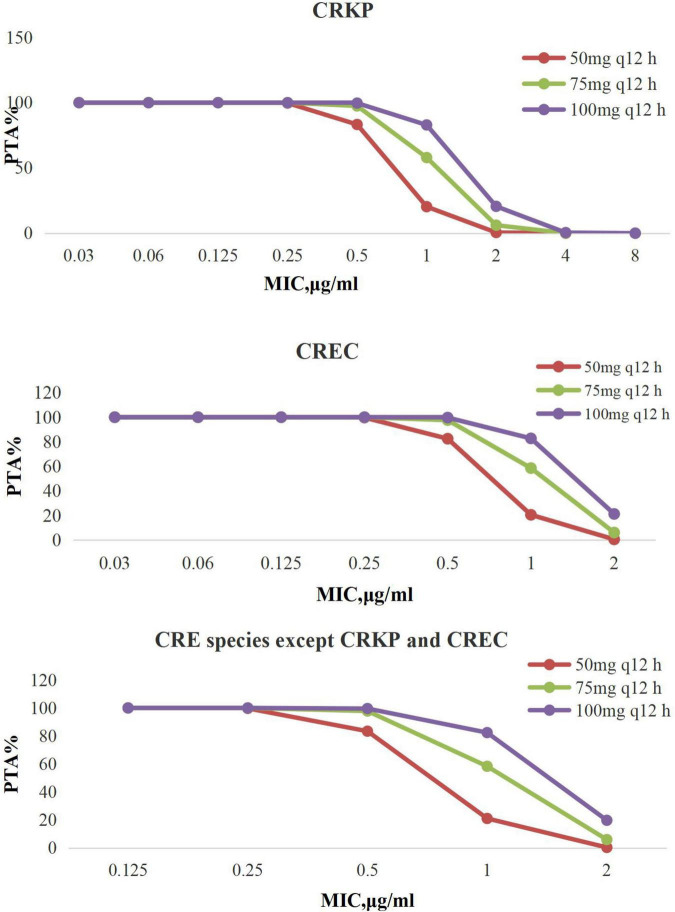
PTA against CRE at MICs from 0.03 to 8 mg/L for tigecycline. PTA, probability target attainment; MIC, minimum inhibitory concentration; CRE, carbapenem-resistant Enterobacterales; CRKP, carbapenem-resistant *Klebsiella pneumoniae*; CREC, carbapenem-resistant *Escherichia coli.*

Targets of AUC_24_/MIC > 6.96 are shown in Figure 1. Tigecycline following administration of 50 mg every 12 h, 75 mg every 12 h, and 100 mg every 12 h achieved > 90% PTAs when MIC was from 0.03 to 8 μg/mL.

**FIGURE 2:**
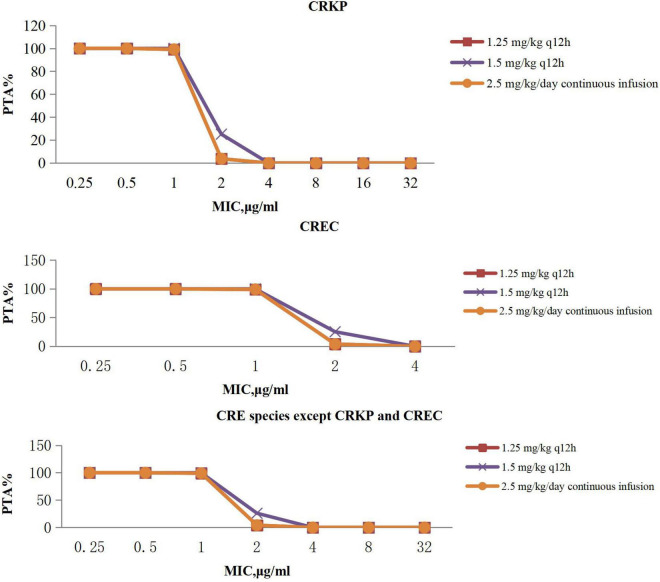
PTA against CRE at MICs from 0.25 to 32 mg/L for polymyxin B. PTA, probability target attainment; MIC, minimum inhibitory concentration; CRE, carbapenem-resistant Enterobacterales; CRKP, carbapenem-resistant *Klebsiella pneumoniae*; CREC, carbapenem-resistant *Escherichia coli*.

The PTAs for polymyxin B regimens at specific MICs with targets of AUC/MIC ≥ 50 are shown in Figure 2. Polymyxin B following administration of 1.25 mg/kg every 12 h, 1.5 mg/kg every 12 h, and 2.5 mg/kg per day continuous infusion achieved > 90% PTAs when MIC was 1 μg/mL with CRE. No regimen achieved a 90% PTA with an MIC of 2 μg/mL.

**FIGURE 3:**
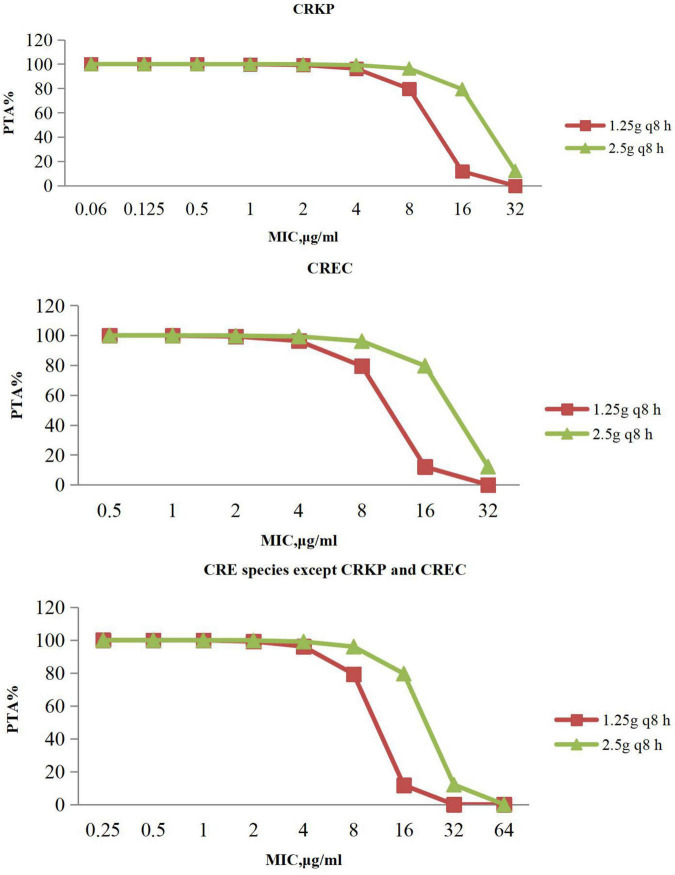
PTA against CRE at MICs from 0.06 to 64 mg/L for ceftazidime/avibactam. PTA, probability target attainment; MIC, minimum inhibitory concentration; CRE, carbapenem-resistant Enterobacterales; CRKP, carbapenem-resistant *Klebsiella pneumoniae*; CREC, carbapenem-resistant *Escherichia coli.*

The PTAs for ceftazidime/avibactam regimens at specific MICs with targets of 50% fT > MIC are shown in Figure 3. Ceftazidime/avibactam following administration of 1.25 g every 8 h, 2.5 g every 8 h achieved > 90% PTAs when MIC was 4 μg/mL, 8 μg/mL with CRE. No regimen of ceftazidime/avibactam achieved a 90% PTA with an MIC of 16 μg/mL with CRE.

### Cumulative Fraction of Response

Tables 5, 6 show the CFR values for each antibiotic regimen based on the Monte Carlo simulations against CRE. As for CFR values of three antimicrobials, ceftazidime/avibactam achieved the lowest CFR values; the highest CFR value was 77.42%. For tigecycline and ceftazidime/avibactam, with simulated regimen improvement, the CFR values were both increased; the lowest CFR of tigecycline values was 73.42%. It is worth noting that the CFR values of polymyxin B were neither very low nor very high; the lowest CFR value of polymyxin B was 80.89%; the most aggressive dosage of 1.5 mg/kg every 12 h provided CFR value of 82.69% against CRE.

**TABLE 5:** CFR values for three antibiotics against CRE.

Antimicrobials	Dosing regimens	CFR (%)
Tigecycline	50 mg every 12 h	73.42
	75 mg every 12 h	85.32
	100 mg every 12 h	91.88
Polymyxin B	1.25 mg/kg every 12 h	81.14
	1.5 mg/kg every 12 h	82.69
	2.5 mg/kg per day continuous infusion	80.89
Ceftazidime/avibactam	1.25 g every 8 h	66.59
	2.5 g every 8 h	77.42

*CFR, cumulative fraction of response; CRE, carbapenem-resistant Enterobacterales.*

**TABLE 6:** CFR values for three antibiotics against CRE.

Antimicrobials	CRE	Dosing regimens	CFR (%)
Tigecycline	CRKP	50 mg every 12 h	69.22
		75 mg every 12 h	83.12
		100 mg every 12 h	91.15
	CREC	50 mg every 12 h	91.45
		75 mg every 12 h	95.53
		100 mg every 12 h	96.77
	CRE species except CRKP and CREC	50 mg every 12 h	85.14
		75 mg every 12 h	92.53
		100 mg every 12 h	95.62
Polymyxin B	CRKP	1.25 mg/kg every 12 h	82.84
		1.5 mg/kg every 12 h	86
		2.5 mg/kg per day continuous infusion	82.79
	CREC	1.25 mg/kg every 12 h	82.6
		1.5 mg/kg every 12 h	86.05
		2.5 mg/kg per day continuous infusion	82.3
	CRE species except CRKP and CREC	1.25 mg/kg every 12 h	77.05
		1.5 mg/kg every 12 h	79.99
		2.5 mg/kg per day continuous infusion	76.31
Ceftazidime/avibactam	CRKP	1.25 g every 8 h	82.48
		2.5 g every 8 h	91.78
	CREC	1.25 g every 8 h	67.79
		2.5 g every 8 h	86.33
	CRE species except CRKP and CREC	1.25 g every 8 h	19.63
		2.5 g every 8 h	29.12

*CFR, cumulative fraction of response; CRE, carbapenem-resistant Enterobacterales; CRKP, carbapenem-resistant Klebsiella pneumoniae; CREC, carbapenem-resistant Escherichia coli.*

## Discussion

Ceftazidime/avibactam is a novel β-lactam/β-lactamase inhibitor combination against CRE that inactivates Ambler class A, class C, and some class D β-lactamase–producing pathogens, including those producing *Klebsiella pneumoniae* carbapenemase and *OXA-48* carbapenemases, but not metallo-β-lactamases (Li et al., 2019), and it has improved survival in multidrug-resistant Gram-negative bacilli infections (Shields et al., 2016, 2017; Temkin et al., 2017; Tumbarello et al., 2019; Clerici et al., 2021). For treatment of all CRE, tigecycline, which is a novel antimicrobial agent with *in vitro* activity against most Gram-positive and Gram-negative pathogens, is mainly used for treatment of complicated skin, soft tissue, and intra-abdominal infections in adults (Babinchak et al., 2005; Ellis-Grosse et al., 2005; Pankey, 2005; Bhavnani et al., 2012; Bodmann et al., 2012). Polymyxin B is considered as the last line of defense against drug-resistant bacteria (Li et al., 2006; Zavascki et al., 2007; Landman et al., 2008; Yu et al., 2017; Nang et al., 2021). Our study analyzed the CRE data of the BRICS to evaluate the effectiveness of the three most commonly used antibacterial for BSIs with CRE in different dosing regimens using Monte Carlo simulations to model *in vivo* antibiotic pharmacodynamics, in the hope that empirical administration will help improve the survival rate of patients.

Ceftazidime/avibactam clinical breakpoints of susceptible MIC ≤ 8 mg/L have been assigned to CRE by CLSI, and the breakpoints of susceptible MIC ≤ 2 mg/L for tigecycline and polymyxin B were assigned to CRE by the US Food and Drug Administration and European Committee on Antimicrobial Susceptibility Testing.

From Tables 3, 4, it could be known that 334 strains were sensitive to ceftazidime/avibactam in CRE, with a susceptibility rate of 75.06% (334/445), which was in line with the literature that the susceptibility rate of ceftazidime/avibactam was 75.0% (Zou et al., 2020), but it was higher than the results reported in 2020 [published by the China Antimicrobial Surveillance Network (CHINET) Study Group, the susceptibility of ceftazidime/avibactam against CRE was 61.4%] (Han et al., 2020); it could be attributed to the strict control of the application of antibacterial recent years. However, our research also revealed that the current MIC_50_ and MIC_90_ of ceftazidime/avibactam against CRE are significantly different with the literature reported (8 vs. 2 mg/L, 16 vs. 32 mg/L) (Han et al., 2020). This phenomenon needs further research. We also found that the MIC of CRE to ceftazidime/avibactam is up to 64 μg/mL, and high MIC of CRE accounts for a high proportion; for example, the percentage of MIC such as 32 μg/mL in other CRE species except CRKP and CREC is as high as 69.49%. This also explains why the CFR of ceftazidime/avibactam is low, which suggests that we empirically apply ceftazidime/avibactam to treat BSIs caused by other CREs and should be used cautiously.

Ceftazidime/avibactam PTA at MIC ≤ 8 and 16 mg/L ranged from 96.01 to 100% and 79.6–79.33% with the dosage of 2.5 g every 8 h, respectively; a similar finding has been observed in adults with complicated intra-abdominal infections, complicated urinary tract infections, and nosocomial pneumonia (Das et al., 2019). PTA was lower with the dosage of 1.25 g every 8 h, but still with high target attainment (>95%) against MICs ≤ 4 mg/L. It was a limitation that the study lacked the enzymes of CRE, which reminded us that we should detect the enzymes produced by CRE of ceftazidime/avibactam-resistant in future work, so as to provide more targeted recommendations for clinical medication.

We also investigated that polymyxin B and tigecycline showed excellent antibacterial activity against CRE strains; 612 strains were sensitive to polymyxin B, with a susceptibility rate of 94.15% (612/650); 640 strains were sensitive to tigecycline, with a susceptibility rate of 99.07% (640/646). The findings were consistent with the literature published by the CHINET Study Group (the susceptibility rates were 95.8 and 98.4% for polymyxin B and tigecycline, respectively) (Han et al., 2020). The data in the study were from the BRICS, covering most provinces in China, and the resistance of CRE was basically consistent with the relevant literature about the resistance of bacteria in China. It truly reflected the resistance of CRE in China, and it has a very high reference value.

For treatment of all CRE, tigecycline achieved the optimal CFRs (>90%) when tigecycline was given 100 mg every 12 h; particularly, it can achieve the satisfactory CFR values for CREC given any dosage regimen, which were in line with the literature that in their response to the high-dose tigecycline (200 mg followed by 100 mg every 12 h), *E. coli* and *K. pneumoniae* showed CFRs greater than 90% (Wang et al., 2020). Our study is consistent with literature reports, when MIC was 1 μg/mL; the PTAs of standard dosing for CRKP, CREC, and other CRE species were 29.84, 29.86, and 28.39%, whereas the other regimen (100 mg every 12 h) PTA was ≥ 88%.

It is worth noting that MIC has a tendency to increase, and the highest MIC of CPKP to tigecycline had reached 8 μg/mL; strains with MIC as high as 2 μg/mL were also found in CREC and other CRE species. Studies have shown that when the MIC is 1 μg/mL, the conventional dosage of tigecycline is worthy of questions (Silvestri and van Saene, 2010), because peak serum levels of tigecycline are low (0.63–1.4 mg/mL) after standard dosing (100 mg followed by 50 mg every 12 h) due to its rapid movement from the bloodstream into tissues after administration (Yamashita et al., 2014), and another study showed that a high-dose tigecycline regimen (200 mg followed by 100 mg every 12 h) was a reasonable strategy for BSIs and other severe infections by CRE (Tumbarello et al., 2018). In general, the CFRs of tigecycline were higher, but because of a lack of exact PK/PD target in BSIs, we still have a suspicion about the efficacy of high-dose tigecycline regimen for use in BSIs with CRE; more prospective studies are needed to determine the clinical benefits of high-dose tigecycline for BSIs with CRE.

Polymyxin B PTA at MIC ≤ 1 mg/L showed excellent target attainment (>98%) at any dosage, whereas PTAs ranged from 3.78 to 25.97% at MIC 2 mg/L. For CRKP and CREC, the CFRs of all administration regimens of polymyxin B could reach 80% or more, and our research showed that polymyxin B could achieve moderate results under majority of conventional dosing regimens, whereas dosing regimens with a CFR between 80 and 90% were regarded as providing moderate probabilities of treatment success (Bradley et al., 2003). For other CRE species, the CFRs ranged from 76.31 to 79.99%, with no administration regimen achieving 90%. However, it is important to note that polymyxin poses a risk of nephrotoxicity (Vattimo M de et al., 2016; Liu et al., 2021; Zeng et al., 2021), especially when administered in large dosage. Data indicated that the tolerated maximum dosage of polymyxin B is 3 mg/kg per day (Liu et al., 2021), although the maximum dosage of polymyxin B is the most effective of all regimens according to simulation; attention should be paid to monitoring renal function when applied.

Monte Carlo simulation was applied in this study to predict the efficacy of three different drug administration regimens in the CRE BSI, without combining the host status, such as combination medication, whether there was hypoproteinemia, and so on, which will lead to different clinical results. In the future, more prospective studies are still needed to evaluate the therapeutic effects of the aforementioned dosing regimens.

## Conclusion

Our study indicates that tigecycline and polymyxin B regimens have high CFR value of BSIs caused by CRE; ceftazidime/avibactam achieved the lowest CFR values among three antimicrobials. Tigecycline regimens were more effective against CRE than the other two antibiotics. For tigecycline and ceftazidime/avibactam, with simulated regimen improvement, the CFR values were both increased. We suggest that measurement of MICs and individualized therapy should be considered together to achieve the optimal drug exposure. In particular, PK and PD modeling based on local antimicrobial resistance data can provide valuable guidance for clinicians for the administration of empirical antibiotic treatments for BSIs.

## Data Availability Statement

The original contributions presented in the study are included in the article/supplementary material, further inquiries can be directed to the corresponding author/s.

## Author Contributions

YJ and YX were responsible for the study conception and design. DZ drafted the manuscript. GY, CS, JJ, CY, PW, ZL, and JW searched the literature. All authors contributed to the article and approved the submitted version.

## Conflict of Interest

The authors declare that the research was conducted in the absence of any commercial or financial relationships that could be construed as a potential conflict of interest.

## Publisher’s Note

All claims expressed in this article are solely those of the authors and do not necessarily represent those of their affiliated organizations, or those of the publisher, the editors and the reviewers. Any product that may be evaluated in this article, or claim that may be made by its manufacturer, is not guaranteed or endorsed by the publisher.

## References

[B1] Averbuch D. Tridello G. Hoek J. Mikulska M. Akan H. Yaòez San Segundo L. (2017). Antimicrobial resistancein gram-negative rods causing bacteremia in hematopoietic stem cell transplant recipients: intercontinental prospective study of the infectious diseases working party of the European bone marrow transplantation group. *Clin. Infect. Dis.* 65 1819–1828. 10.1093/cid/cix646 29020364

[B2] Babinchak T. Ellis-Grosse E. Dartois N. Rose G. M. Loh E. Tigecycline 301 Study Group (2005). The efficacy and safety of tigecycline for the treatment of complicated intra-abdominal infections: analysis of pooled clinical trial data. *Clin. Infect. Dis.* 41(Suppl. 5) S354–S367. 10.1086/431676 16080073

[B3] Bensman T. J. Wang J. Jayne J. Fukushima L. Rao A. P. D’Argenio D. Z. (2017). Pharmacokinetic-pharmacodynamic target attainment analyses to determineoptimal dosing of ceftazidime-avibactamforthe treatment of acute pulmonary exacerbations in patients with cystic fibrosis. *Antimicrob. Agents Chemother.* 61:e00988-17. 10.1128/AAC.00988-17 28784670 PMC5610479

[B4] Bhavnani S. M. Rubino C. M. Hammel J. P. Forrest A. Dartois N. Cooper C. A. (2012). Pharmacological and patient-specific response determinants in patients with hospital-acquired pneumonia treated with tigecycline. *Antimicrob. Agents Chemother.* 56 1065–1072. 10.1128/AAC.01615-10 22143524 PMC3264202

[B5] Bodmann K. F. Heizmann W. R. von Eiff C. Petrik C. Löschmann P. A. Eckmann C. (2012). Therapy of 1,025 severely ill patients with complicated infections in a German multicenter study: safety profile and efficacy of tigecycline in different treatment modalities. *Chemotherapy.* 58 282–294. 10.1159/000342451 23052187

[B6] Bradley J. S. Dudley M. N. Drusano G. L. (2003). Predicting efficacy of antiinfectives with pharmacodynamics and Monte Carlo simulation. *Pediatr. Infect. Dis J.* 22 982–995. 10.1097/01.inf.0000094940.81959.14 14614372

[B7] Centers for Disease Control and Prevention (2013). *Antibiotic Resistance Threats in the United States,2013[EB/OL].* Available online at: http://www.cdc.gov/drugresistance/threat-report-2013/pdf/ar-threats-2013-508.pdf (accessed October 9, 2020)

[B8] Chen H. Y. Jean S. S. Lee Y. L. Lu M. C. Ko W. C. Liu P. Y. (2021). Carbapenem-resistant enterobacterales in long-term care facilities: a global and narrative review. *Front. Cell. Infect. Microbiol.* 11:601968. 10.3389/fcimb.2021.601968 33968793 PMC8102866

[B9] Clerici D. Oltolini C. Greco R. Ripa M. Giglio F. Mastaglio S. (2021). The place in therapy of ceftazidime/avibactam and ceftolozane/tazobactam in hematological patients with febrile neutropenia. *Int. J. Antimicrob. Agents* 57:106335. 10.1016/j.ijantimicag.2021.106335 33838223

[B10] CLSI (2019). *Performance Standards for Antimicrobial Susceptibility Testing, Informational Supplement. CLSI Document M100-S29*, 29th Edn. Wayne, PA: Clinical and Laboratory Standards Institute.

[B11] Das S. Li J. Riccobene T. Carrothers T. J. Newell P. Melnick D. (2019). Dose selection and validation for ceftazidime-avibactam in adults with complicated intra-abdominal infections, complicated urinary tract infections, and nosocomial pneumonia. *Antimicrob. Agents Chemother.* 63:e02187-18. 10.1128/AAC.02187-18 30670413 PMC6437548

[B12] Dautzenberg M. J. Wekesa A. N. Gniadkowski M. Antoniadou A. Giamarellou H. Petrikkos G. L. (2015). The association between colonization with carbapenemase-producing Enterobacteriaceae and overall ICU mortality: an observational cohort study. *Crit. Care Med.* 43 1170–1177. 10.1097/CCM.0000000000001028 25882764 PMC4431676

[B13] Drusano G. L. Preston S. L. Hardalo C. Hare R. Banfield C. Andes D. (2001). Use of preclinical data for selection of a phase II/III dose for evernimicin and identification of a preclinical MIC breakpoint. *Antimicrob. Agents Chemother.* 45 13–22. 10.1128/AAC.45.1.13-22.2001 11120938 PMC90233

[B14] El-Gamal M. I. Brahim I. Hisham N. Aladdin R. Mohammed H. Bahaaeldin A. (2017). Recent updates of carbapenem antibiotics. *Eur. J. Med. Chem.* 131 185–195. 10.1016/j.ejmech.2017.03.022 28324783

[B15] Ellis-Grosse E. J. Babinchak T. Dartois N. Rose G. Loh E. Tigecycline 300 cSSSI Study Group (2005). The efficacy and safety of tigecycline in the treatment of skin and skin-structure infections: results of 2 double-blind phase 3 comparison studies with vancomycin-aztreonam. *Clin. Infect. Dis.* 41(Suppl. 5) S341–S353. 10.1086/431675 16080072

[B16] Falagas M. E. Tansarli G. S. Karageorgopoulos D. E. Vardakas K. Z. (2014). Deaths attributable to carbapenem-resistant Enterobacteriaceae infections. *Emerg. Infect. Dis.* 20 1170–1175. 10.3201/eid2007.121004 24959688 PMC4073868

[B17] Han R. Shi Q. Wu S. Yin D. Peng M. Dong D. (2020). Dissemination of carbapenemases (KPC, NDM, OXA-48, IMP, and VIM) among carbapenem-resistant Enterobacteriaceae isolated from adult and children patients in China. *Front. Cell. Infect. Microbiol.* 10:314. 10.3389/fcimb.2020.00314 32719751 PMC7347961

[B18] Hussein K. Raz-Pasteur A. Finkelstein R. Neuberger A. Shachor-Meyouhas Y. Oren I. (2013). Impact of carbapenem resistance on the outcome of patients’ hospital-acquired bacteraemia caused by *Klebsiella pneumoniae*. *J. Hosp. Infect.* 83 307–313. 10.1016/j.jhin.2012.10.012 23313086

[B19] Landman D. Georgescu C. Martin D. A. Quale J. (2008). Polymyxins revisited. *Clin. Microbiol. Rev.* 21 449–465. 10.1128/CMR.00006-08 18625681 PMC2493081

[B20] Laupland K. B. Church D. L. (2014). Population-based epidemiology and microbiology of community-onset bloodstream infections. *Clin. Microbiol. Rev.* 27 647–664. 10.1128/CMR.00002-14 25278570 PMC4187633

[B21] Lee H. Lee H. (2016). Clinical and economic evaluation of multidrug-resistant *Acinetobacter baumannii* colonization in the intensive care unit. *Infect. Chemother.* 48 174–180. 10.3947/ic.2016.48.3.174 27659440 PMC5047998

[B22] Li J. Lovern M. Green M. L. Chiu J. Zhou D. Comisar C. (2019). Ceftazidime-avibactam population pharmacokinetic modeling and pharmacodynamic target attainment across adult indications and patient subgroups. *Clin. Transl. Sci.* 12 151–163. 10.1111/cts.12585 30221827 PMC6440567

[B23] Li J. Nation R. L. Turnidge J. D. Milne R. W. Coulthard K. Rayner C. R. (2006). Colistin: the re-emerging antibiotic for multidrug-resistant Gram-negative bacterial infections. *Lancet Infect. Dis.* 6 589–601. 10.1016/S1473-3099(06)70580-1 16931410

[B24] Little M. L. Qin X. Zerr D. M. Weissman S. J. (2012). Molecular diversity in mechanisms of carbapenem resistance in paediatric Enterobacteriaceae. *Int. J. Antimicrob. Agents* 39 52–57. 10.1016/j.ijantimicag.2011.09.014 22055532 PMC3237943

[B25] Liu X. Chen Y. Yang H. Li J. Yu J. Yu Z. (2021). Acute toxicity is a dose-limiting factor for intravenouspolymyxin B: a safety and pharmacokinetic study in healthy Chinese subjects. *J. Infect.* 82 207–215. 10.1016/j.jinf.2021.01.006 33453286

[B26] Meatherall B. L. Gregson D. Ross T. Pitout J. D. Laupland K. B. (2009). Incidence, risk factors, and outcomes of *Klebsiella pneumoniae* bacteremia. *Am. J. Med.* 122 866–873. 10.1016/j.amjmed.2009.03.034 19699383

[B27] Miglis C. Rhodes N. J. Avedissian S. N. Kubin C. J. Yin M. T. Nelson B. C. (2018). Population pharmacokinetics of polymyxin B in acutely ill adult patients. *Antimicrob. Agents Chemother.* 62:e01475-17. 10.1128/AAC.01475-17 29311071 PMC5826159

[B28] Mouton J. W. Dudley M. N. Cars O. Derendorf H. Drusano G. L. (2005). Standardization of pharmacokinetic/pharmacodynamic (PK/PD) terminology for anti-infective drugs: an update. *J. Antimicrob. Chemother.* 55 601–607. 10.1093/jac/dki079 15772142

[B29] Nang S. C. Azad M. A. K. Velkov T. Zhou Q. T. Li J. (2021). Rescuing the last-line polymyxins: achievements and challenges. *Pharmacol. Rev.* 73 679–728. 10.1124/pharmrev.120.000020 33627412 PMC7911091

[B30] Neuwirth C. Siébor E. Duez J. M. Péchinot A. Kazmierczak A. (1995). Imipenem resistance in clinical isolates of *Proteus mirabilis* associated with alterations in penicillin-binding proteins. *J. Antimicrob. Chemother.* 36 335–342. 10.1093/jac/36.2.335 8522463

[B31] Pankey G. A. (2005). Tigecycline. *J. Antimicrob. Chemother.* 56 470–480. 10.1093/jac/dki248 16040625

[B32] Patel G. Huprikar S. Factor S. H. Jenkins S. G. Calfee D. P. (2008). Outcomes of carbapenem-resistant *Klebsiella pneumoniae* infection and the impact of antimicrobial and adjunctive therapies. *Infect. Control Hosp. Epidemiol.* 29 1099–1106. 10.1086/592412 18973455

[B33] Rahal J. J. (2008). The role of carbapenems in initial therapy for serious Gram-negative infections. *Crit. Care* 12(Suppl. 4):S5. 10.1186/cc6821 18495062 PMC2391262

[B34] Rubino C. M. Forrest A. Bhavnani S. M. Dukart G. Cooper A. Korth-Bradley J. (2010). Tigecycline population pharmacokinetics in patients with community- or hospital-acquired pneumonia. *Antimicrob. Agents Chemother.* 54 5180–5186. 10.1128/AAC.01414-09 20921315 PMC2981274

[B35] Satlin M. J. Calfee D. P. Chen L. Fauntleroy K. A. Wilson S. J. Jenkins S. G. (2013). Emergence of carbapenem-resistant Enterobacteriaceae as causes of bloodstream infections in patients with hematologic malignancies. *Leuk. Lymphoma* 54 799–806. 10.3109/10428194.2012.723210 22916826

[B36] Satlin M. J. Cohen N. Ma K. C. Gedrimaite Z. Soave R. Askin G. (2016). Bacteremia due to carbapenem-resistant Enterobacteriaceae in neutropenic patients with hematologic malignancies. *J. Infect.* 73 336–345. 10.1016/j.jinf.2016.07.002 27404978 PMC5026910

[B37] Shields R. K. Nguyen M. H. Chen L. Press E. G. Potoski B. A. Marini R. V. (2017). Ceftazidime-avibactam is superior to other treatment regimens against carbapenem-resistant *Klebsiella pneumoniae* bacteremia. *Antimicrob. Agents Chemother.* 61:e00883-17. 10.1128/AAC.00883-17 28559250 PMC5527595

[B38] Shields R. K. Potoski B. A. Haidar G. Hao B. Doi Y. Chen L. (2016). Clinical outcomes, drug toxicity, and emergence of ceftazidime-avibactam resistance among patients treated for carbapenem-resistant Enterobacteriaceae infections. *Clin. Infect. Dis.* 63 1615–1618. 10.1093/cid/ciw636 27624958 PMC5146720

[B39] Sievert D. M. Ricks P. Edwards J. R. Schneider A. Patel J. Srinivasan A. (2013). Antimicrobial-resistant pathogens associated with healthcare-associated infections: summary of data reported to the National Healthcare Safety Network at the Centers for Disease Control and Prevention, 2009-2010. *Infect. Control Hosp. Epidemiol.* 34 1–14. 10.1086/668770 23221186

[B40] Silvestri L. van Saene H. K. (2010). Hospital-acquired infections due to gram-negative bacteria. *N. Engl. J. Med.* 363 1482–1484. 10.1056/NEJMc1006641 20925554

[B41] Temkin E. Torre-Cisneros J. Beovic B. Benito N. Giannella M. Gilarranz R. (2017). Ceftazidime-avibactam as salvage therapy for infections caused by carbapenem-resistant organisms. *Antmicrob. Agents Chemother.* 61:e01964-16. 10.1128/AAC.01964-16 27895014 PMC5278727

[B42] Thamlikitkul V. Dubrovskaya Y. Manchandani P. Ngamprasertchai T. Boonyasiri A. Babic J. T. (2016). Dosing and pharmacokinetics of polymyxin B in patients with renal insufficiency. *Antimicrob. Agents Chemother.* 61:e01337-16. 10.1128/AAC.01337-16 27799209 PMC5192162

[B43] Tumbarello M. Losito A. R. Giamarellou H. (2018). Optimizing therapy in carbapenem-resistant Enterobacteriaceae infections. *Curr. Opin. Infect. Dis.* 31 566–577. 10.1097/QCO.0000000000000493 30379732

[B44] Tumbarello M. Trecarichi E. M. Corona A. De Rosa F. G. Bassetti M. Mussini C. (2019). Efficacy of ceftazidime-avibactam salvage therapy in patients with infections caused by *Klebsiella pneumoniae* carbapenemase-producing *K. Pneumoniae*. *Clin. Infect. Dis.* 68 355–364. 10.1093/cid/ciy492 29893802

[B45] Tumbarello M. Viale P. Viscoli C. Trecarichi E. M. Tumietto F. Marchese A. (2012). Predictors of mortality in bloodstream infections caused by *Klebsiella pneumoniae* carbapenemase-producing *K. pneumoniae*: importance of combination therapy. *Clin. Infect. Dis.* 55 943–950. 10.1093/cid/cis588 22752516

[B46] Vasoo S. Barreto J. N. Tosh P. K. (2015). Emerging issues in gram-negative bacterial resistance: an update for the practicing clinician. *Mayo Clin. Proc.* 90 395–403. 10.1016/j.mayocp.2014.12.002 25744116

[B47] Vattimo M de F. Watanabe M. da Fonseca C. D. Neiva L. B. Pessoa E. A. Borges F. T. (2016). Polymyxin B nephrotoxicity: from organ to cell damage. *PLoS One* 11:e0161057. 10.1371/journal.pone.0161057 27532263 PMC4988638

[B48] Wang C. Hao W. Jin Y. Shen C. Wang B. (2020). Pharmacokinetic/pharmacodynamic modeling of seven antimicrobials for empiric treatment of adult bloodstream infections with gram-negative bacteria in China. *Microb Drug Resist.* 26 1559–1567. 10.1089/mdr.2019.0152 31794682

[B49] Wu Y. E. Xu H. Y. Shi H. Y. van den Anker J. Chen X. Y. Zhao W. (2020). Carbapenem-resistant Enterobacteriaceae bloodstream infection treated successfully with high-dose meropenem in a preterm neonate. *Front. Pharmacol.* 11:566060. 10.3389/fphar.2020.566060 33041807 PMC7518023

[B50] Yamashita N. Matschke K. Gandhi A. Korth-Bradley J. (2014). Tigecycline pharmacokinetics, tolerability, safety, and effect on intestinal microflora in healthy Japanese male subjects. *J. Clin. Pharmacol.* 54 513–519. 10.1002/jcph.236 24243316

[B51] Yu Y. Fei A. Wu Z. Gao C. Pan S. (2017). Intravenous polymyxins: revival with puzzle. *Biosci. Trends.* 11 370–382. 10.5582/bst.2017.01188 28904326

[B52] Zarkotou O. Pournaras S. Tselioti P. Dragoumanos V. Pitiriga V. Ranellou K. (2011). Predictors of mortality in patients with bloodstream infections caused by KPC-producing *Klebsiella pneumoniae* and impact of appropriate antimicrobial treatment. *Clin. Microbiol. Infect.* 17 1798–1803. 10.1111/j.1469-0691.2011.03514.x 21595793

[B53] Zavascki A. P. Goldani L. Z. Li J. Nation R. L. (2007). Polymyxin B for the treatment of multidrug-resistant pathogens: a critical review. *J. Antimicrob. Chemother.* 60 1206–1215. 10.1093/jac/dkm357 17878146

[B54] Zeng H. Zeng Z. Kong X. Zhang H. Chen P. Luo H. (2021). Effectiveness and nephrotoxicity of intravenous polymyxin B in Chinese patients with MDR and XDR nosocomial pneumonia. *Front. Pharmacol.* 11:579069. 10.3389/fphar.2020.579069 33613276 PMC7892461

[B55] Zhu W. Chu Y. Zhang J. Xian W. Xu X. Liu H. (2020). Pharmacokinetic and pharmacodynamic profiling of four antimicrobials against *Acinetobacter baumannii* infection. *Microb. Pathog.* 138:103809. 10.1016/j.micpath.2019.103809 31634531

[B56] Zou C. Wei J. Shan B. Chen X. Wang D. Niu S. (2020). In vitro activity of ceftazidime-avibactam and aztreonam-avibactam against carbapenem-resistant Enterobacteriaceae isolates collected from three secondary hospitals in Southwest China between 2018 and 2019. *Infect. Drug Resist.* 13 3563–3568. 10.2147/IDR.S273989 33116675 PMC7567573

